# A versatile palindromic amphipathic repeat coding sequence horizontally distributed among diverse bacterial and eucaryotic microbes

**DOI:** 10.1186/1471-2164-11-430

**Published:** 2010-07-13

**Authors:** Kerstin Röske, Mark F Foecking, Shibu Yooseph, John I Glass, Michael J Calcutt, Kim S Wise

**Affiliations:** 1 Saxony Academy of Sciences Leipzig, D-04107 Leipzig, Germany; 2 Department of Molecular Microbiology and Immunology, University of Missouri, Columbia MO 65211, USA; 3 J. Craig Venter Institute, 10355 Science Center Drive, San Diego, CA 92121,USA; 4 J. Craig Venter Institute, 9704 Medical Center Drive, Rockville, MD 20850, USA; 5 Department of Veterinary Pathobiology, University of Missouri, Columbia MO 65211, USA; 6 Technische Universität Dresden, Dresden, Germany

## Abstract

**Background:**

Intragenic tandem repeats occur throughout all domains of life and impart functional and structural variability to diverse translation products. Repeat proteins confer distinctive surface phenotypes to many unicellular organisms, including those with minimal genomes such as the wall-less bacterial monoderms, *Mollicutes*. One such repeat pattern in this clade is distributed in a manner suggesting its exchange by horizontal gene transfer (HGT). Expanding genome sequence databases reveal the pattern in a widening range of bacteria, and recently among eucaryotic microbes. We examined the genomic flux and consequences of the motif by determining its distribution, predicted structural features and association with membrane-targeted proteins.

**Results:**

Using a refined hidden Markov model, we document a 25-residue protein sequence motif tandemly arrayed in variable-number repeats in ORFs lacking assigned functions. It appears sporadically in unicellular microbes from disparate bacterial and eucaryotic clades, representing diverse lifestyles and ecological niches that include host parasitic, marine and extreme environments. Tracts of the repeats predict a malleable configuration of recurring domains, with conserved hydrophobic residues forming an amphipathic secondary structure in which hydrophilic residues endow extensive sequence variation. Many ORFs with these domains also have membrane-targeting sequences that predict assorted topologies; others may comprise reservoirs of sequence variants. We demonstrate expressed variants among surface lipoproteins that distinguish closely related animal pathogens belonging to a subgroup of the *Mollicutes*. DNA sequences encoding the tandem domains display dyad symmetry. Moreover, in some taxa the domains occur in ORFs selectively associated with mobile elements. These features, a punctate phylogenetic distribution, and different patterns of dispersal in genomes of related taxa, suggest that the repeat may be disseminated by HGT and intra-genomic shuffling.

**Conclusions:**

We describe novel features of PARCELs (**P**alindromic **A**mphipathic **R**epeat **C**oding **EL**ements), a set of widely distributed repeat protein domains and coding sequences that were likely acquired through HGT by diverse unicellular microbes, further mobilized and diversified within genomes, and co-opted for expression in the membrane proteome of some taxa. Disseminated by multiple gene-centric vehicles, ORFs harboring these elements enhance accessory gene pools as part of the "mobilome" connecting genomes of various clades, in taxa sharing common niches.

## Background

Intragenic repeats encoding recurrent protein domains are abundant, diverse and profoundly affect protein structure and function in a broad assortment of cellular processes and diseases [1-4]. Repeat proteins typically contain tandemly arrayed, redundant sequence patterns of 20-40 amino acids, and many embody extended architectures (structured or disordered) with a capacity to bind natural ligands with a broad range of specificities [4,5], a property recently exploited to engineer specific binding in synthetic counterparts [5,6]. While most abundant in higher eucaryotes [4,7,8], repeat proteins also occur widely among surface proteins of unicellular microbes [8,9], where they mediate interactions within complex environments and communities, and confer variable phenotypes promoting niche adaptation. Surface membrane proteins with repeating sequence motifs abound even among minimalist organisms such as *Mollicutes *(phylum Tenericutes, herein also termed mycoplasmas) a clade of wall-less monoderms with minimal-size, low G+C genomes and parasitic lifestyles. These products are most commonly encoded by families of accessory genes [10,11] specific to a particular clade or individual taxon, in which distinctive repeats are encoded by individual genes [12-14]. As in many parasitic microbes [8], most repeat proteins of *Mollicutes *contain highly similar sequences repeated within an ORF. A notable exception to this theme is a tandem repeat pattern of 25 residues, initially reported in the LppQ lipoprotein (LP) expressed on the surface of the bovine pathogen *Mycoplasma mycoides *subsp *mycoides *small colony biotype (*Mmm *SC) and later in another surface LP of this organism [15,16]. Repeats of this category contain conserved amino acid residues but show considerable sequence variation among individual copies, analogous to other classes of well-studied repeat proteins described [4,5]. Moreover, whereas ORFs with this pattern were initially limited to a narrow phylogenetic group of *Mollicutes *termed the "*Mycoplasma mycoides *cluster" of ruminant pathogens [17,18], counterparts in the genome of the caprine pathogen *Mycoplasma agalactiae *(from a distinct phylogenetic clade of the *Mollicutes*) have been recently reported, and are proposed to represent gene exchange through HGT between these disparate taxa sharing common hosts [19]. The distinctive sequence diversity in this repeat pattern, its demonstrated expression in two known surface membrane proteins, and the prospect that the coding sequence is disseminated horizontally prompted its further examination as a model for the acquisition of a versatile coding module contributing to proteomic diversity.

Analyses of sequence databases by others have independently identified two motifs that correspond indirectly to the sequence pattern described, each defined by a profile hidden Markov model (HMM) [20]: (i) DUF (domain of unknown function) 285, termed "mycoplasma protein of unknown function" or "protein of unknown function, lipoprotein predicted" (Pfam [21]: 03382; InterPro [22]: IPR005046) and (ii) "bacterial surface protein 26-residue repeat" (TIGRFAM [23]: TIGR02167; InterPro: IPR011889). Inspection showed that while these HMMs approximate and include the original sequence pattern, neither description defines a uniform unit repeat, the predicted nature of the repeated domain, accurate features of many ORFs now identified, or the phylogenetic groups represented by these motifs. Repeated interrogations of protein sequence databases [24] revealed that these HMMs occur in ORFs from an increasingly broad range of organisms, including remotely-related clades of bacteria and, more recently, selected lineages of unicellular eucaryotes and metagenomic samples from the marine environment. These observations prompted us to characterize the distribution and predicted properties of the motif in order to understand its possible exploitation as a genetic module, and to ascribe more informative attributes to this broadly disseminated DUF.

Here we refine the definition of this repeat sequence motif and report its dispersal among genomes representing diverse parasitic and environmental bacteria, unicellular eucaryotes, and samples of the marine metagenome. Using several examples, we describe its predicted secondary structure and sequence variation, the unexpected dyad symmetry of its coding sequences, and its occurrence in ORFs encoding assorted types of membrane proteins. We experimentally verify its contribution to phenotypic diversity in expressed surface proteins of two closely related mycoplasmal pathogens. By comparing the complete genome sequences of related taxa from lineages representing multiple, disparate phylogenetic clades, we further document chromosomal distributions of the motif and its association with mobility elements that suggest transfer by HGT and intra-genomic shuffling in diverse evolutionary settings. Through these studies we define the protein domain and coding sequence PARCEL (**P**alindromic **A**mphipathic **R**epeat **C**oding **EL**ement), having newly-recognized attributes of structural versatility and adaptive variation, with a capacity to expand and diversify repertoires of membrane proteins in some taxa. We reason that this repeat sequence has been transferred by multiple pathways as part of the pervasive mobilome that actively shapes the genomic makeup of selected microorganisms.

## Results and Discussion

### Dispersal of a tandemly arrayed repeat protein sequence in the genomes of unicellular microbes from diverse phylogenetic clades and ecological niches

**Figure 1:**
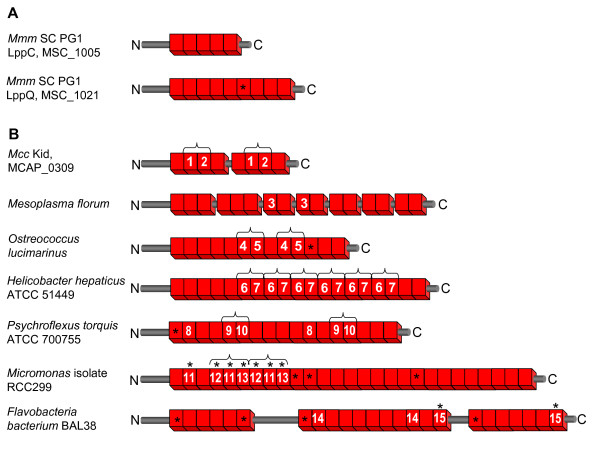
**Examples of tandem repeats in assorted ORFs**. Cartoons show representative patterns of tandemly arrayed HMM domains (red boxes) within ORFs (rods). Domains of unique sequence (unmarked) or shared identical sequence (similarly-numbered) are indicated. Asterisks (*) denote non-canonical domains with fewer or more than 25 residues. (A) The most prevalent pattern comprises uninterrupted tracts of tandem repeats, with each unit having a unique sequence, as represented by two expressed mycoplasmal LPs (LppC and LppQ). (B) Less common patterns include tracts comprising repeated blocks of domains (indicated by brackets), occasionally separated by spacer regions of unrelated sequence (rods between blocks). Depictions are not to scale and do not show the precise location of tracts in an ORF. ORFs representing the respective taxa listed include: *Mmm *SC PG1 (LppC), gi|42561519|ref|NP_975970.1|; *Mmm *SC PG1 (LppQ), gi|42561534|ref|NP_975985.1|; *Mcc *Kid (MCAP_0309), gi|83319306|ref|YP_424295.1|; *Mesoplasma florum* L1, gi|50365263|ref|YP_053688.1|; *Ostreococcus lucimarinus *CCE9901, gi|145356136|ref|XP_001422294.1|; *Helicobacter hepaticus *ATCC 51449, gi|32265549|ref|NP_859581.1|; *Psychroflexus torquis *ATCC 700755, gi|91218343|ref|ZP_01255287.1|; *Micromonas sp*. RCC299, gi|226517946|gb|ACO63939.1|; *Flavobacteria bacterium *BAL38, gi|126663436|ref|ZP_01734433.1|

To establish an operational motif we first constructed an HMM based on a training dataset of ORFs from genomes of *Mollicutes *that contained a previously reported 25-residue amino acid sequence pattern [15], then refined the HMM using iterations of data sets expanded from successive searches of the non-redundant protein sequence database [24]. The HMM included sequences from diverse organisms representing very different phylogenetic histories, genomic sizes and G+C contents. We interrogated a recent version of this database (nr; October 30, 2009; 9,967,556 sequences) with the HMM to inventory the current set of unique ORFs bearing the motif. ORFs retrieved (Additional file 1, sheet 1) were organized using NCBI taxonomic classifiers [24]. Under the two superkingdoms represented, Bacteria and Eucaryota, the corresponding taxa are organized by genus, species and further identifiers. Individual ORFs of a taxon are denoted by unique identifiers (GI and accession numbers), overall HMM scores and numbers of HMM domains present in each. From our low stringency search, fourteen ORFs with poor HMM scores (E-values > 1) were excluded from consideration (not shown). The remaining 461 unique ORFs retrieved by the HMM span a variety of taxonomic groups (further detailed below). Nearly all ORFs, irrespective of taxon, contain multiple copies of the HMM domain. When parsed to show the order and location of individual domains (Additional file 1, sheet 2), most ORFs revealed tracts comprising multiple tandem repeats, ranging from two to 59 units in length. Examples are illustrated in Figure 1. This conspicuous feature also pertained to a set of 1736 ORFs (Additional file 2, sheet 1 and sheet 2) retrieved with the HMM from the separate database [24] of non-redundant protein sequences from environmental samples (nr_env; July 6, 2009; 6,028,192 sequences). These ORFs represented individual sequences derived from the Global Ocean Sampling (GOS) project [25] and are not assigned to taxa.

The nature and configuration of individual repeats recognized by the HMM varied among ORFs. The great majority from either database contained tandem arrays of canonical 25-residue repeats, whereas some others contained tracts having intermittent repeats with more or fewer than 25 residues (examples in Figure 1A and 1B). These non-canonical repeats had lower HMM scores; some were prevalent in, and had sequences characteristic of certain lineages, for example the genera *Listeria*, *Lactobacillus, Lactococcus*, and *Enterococcus *(Additional file 1, sheet 2). Infrequently, domains of identical or near-identical sequence recurred in blocks (Figure 1B). This was an exception to the highly variable domain sequences found in most tracts (Figure 1A) and most likely reflects recent duplications known to expand repeat protein domains through strand misalignment during DNA replication [26] or other mechanisms [7]. An additional, distinctive feature observed in a few ORFs was the separation of blocks of tandem repeats by various "spacer" sequences unrelated to the repeating domain. These included short segments sharing similar sequences, or very long regions of unique sequence (Figure 1B). Overall, however, the tandem arrangement of canonical 25-residue units is a hallmark of most ORFs displaying the motif, hence is a defining attribute of this set of repeat proteins.

Neighbor-joining trees of taxa containing the HMM-defined motif revealed its distribution among extraordinarily diverse phylogenetic groups (Additional Files 3 and 4). All taxa identified are unicellular microbes representing two domains of life, Bacteria or Eucaryota, with the great majority residing in the former. Among bacteria (Additional File 3), several major groups are represented, reflecting a wide range of genome sizes and G+C compositions. Eucaryotic taxa (Additional File 4A) are currently represented by only two, deeply-separated lineages belonging to photosynthetic marine microalgae [27-29]: the primary endosymbiotic Prasinophytes (green algae), and the secondary endosymbiotic Stramenopiles (diatoms). In both domains of life, great phylogenetic distances separate clades that harbor the motif, yet it is distributed only sporadically within some clades of closely related taxa. This is best illustrated by a set of 891 bacterial and archaeal taxa having fully sequenced and assembled genomes, scored to indicate the presence or absence of annotated ORFs bearing the motif (Additional file 5). The motif occurs in only 34 of these bacterial taxa, which are distributed among distant branches radiating from deeply rooted, higher order groups. It is represented by only a single taxon in some broad groups, yet appears selectively in subsets of other closely related taxa (e.g., among lineages representing *Lactobacillus*, *Listeria, Helicobacter, Prochlorococcus*, and *Mycoplasma*). The absence of the motif from genomes of the Archaea is conspicuous, but may simply reflect the comparatively small sample of complete genome sequences currently available in this branch. Finally, it is striking that the small (~1 Mb) genomes of some *Mollicutes *show an abundance of ORFs containing the motif (detailed in Additional File 4B); these reside selectively in the *Mycoplasma mycoides *phylogenetic cluster and in a separate branch represented by *M. agalactiae *(as also noted in a recent survey of DUF285-associated ORFs among fully sequenced mycoplasmal genomes [19]). Overall, these patterns of occurrence underscore the markedly punctate distribution [30] of the motif at several phylogenetic levels.

The habitats and lifestyles of motif-containing taxa are also broad-ranging. For example, among environmental bacteria are the abundant marine cyanobacteria (*Prochlorococcus marinus*), extreme hyperhalophiles (*Salinibacter ruber*), and green sulfur bacteria (*Chloroherpeton thalassium*). Parasitic bacteria include human agents that are pathogens (*Listeria monocytogenes*), commensal flora of the gut (*Enterococcus faecalis*, *Coprococcus eutactus, Eubacterium biforme*) and a member of the newly-described phylum *Synergistetes *(*Jonquetella anthropi*) [31]; as well as animal pathogens (*Helicobacter hepaticus*, *Mycoplasma *spp.) and parasitic organisms of plants (*Mesoplasma florum*). The population structures of these taxa differ markedly as well. Whereas obligate host parasites typically have small populations subject to frequent evolutionary bottlenecks [32], some environmental organisms such as the open-ocean prochlorococci have very large populations subject to genome streamlining and reduction [33]. Thus the presence of the motif in bacteria is not restricted to any particular phylogenetic group, environmental niche, lifestyle or population structure. Notably, the photosynthetic eucaryotic taxa harboring the motif are globally dispersed in marine and other aquatic environments. The diatom *Phaeodactylum tricornutum*, and the green algae, *Ostreococcus *spp. and *Micromonas *spp., all have minimal cellular designs, with single plastids and mitochondria, and include the smallest known eucaryotic genome (*O. tauri*). The identification of numerous ORFs in environmental sequences from the GOS project (Additional file 2) further indicates that the motif is richly represented in the marine ecosystem, although unassigned to taxa. Remarkably, almost none of the sequences of individual domains in this database (specifically 69 of 11,149 total) had a partner in the nr database with identical sequence, suggesting a high degree of unexplored sequence variation among the HMM domains represented in the marine environment.

### PARCELs: a family of sequence-variable amphipathic protein domains encoded by tracts of palindromic repeats

We sought to determine additional characteristics of the coding regions corresponding to our HMM as a means to understand their possible structural or functional commonalities. Although most are recognized also by the HMMs DUF285 and TIGR02167 (data not shown), little else is known about these regions or the ORFs harboring them. These ORFs generally were not assigned functional categories nor were recognizable housekeeping genes. For example, only nine of the 406 ORFs listed in Additional file 1 were assigned to COGs, each having ambiguous or anomalous descriptors (data not shown). Further classification of ORFs by approaches such as BLAST was hampered by the nature of the motif, which displayed variation in the sequences of individual repeats, and in the length of their tandem arrays. Nevertheless, inspection of several ORFs revealed that the protein sequences flanking tracts of repeats were often specific to the ORF. We therefore considered the repeats as modular regions embedded in different sequence contexts and examined the predicted properties of representative modules *per se*.

**Figure 2:**
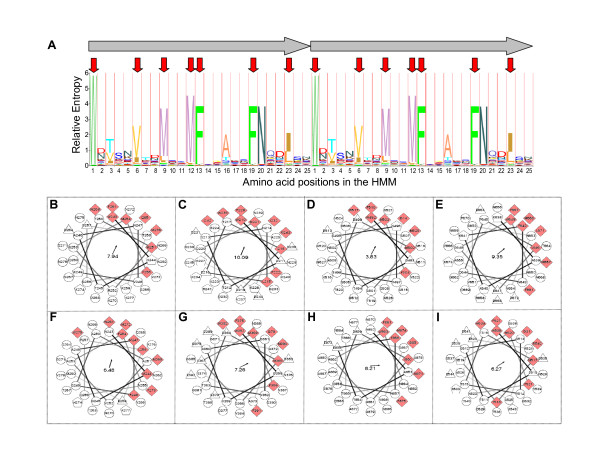
**Predicted protein sequence features of HMM domains**. (A) A protein sequence HMM logo indicates the relative probabilities of amino acid residues (single letter code) at positions within the canonical 25-residue domain. The logo is shown in tandem duplicate (horizontal arrows). Heights of letters correspond to the relative entropy, indicating the prevalence (observed vs. *a priori *expected frequency) of residues at the respective position. Positions of conserved hydrophobic residues occurring periodically in the domains are indicated by red arrows. (B-I) Helical wheel projections of HMM domains from assorted taxa. Sequences of 36 residues (to encompass or bridge individual HMM motifs) represent adjacent, canonical domains. Amino acids are denoted by residue number in the ORF and by hydrophobicity as described in Methods: hydrophobic (red-shaded diamonds), hydrophilic (circles), negatively-charged (triangles) and positively-charged (pentagons). Asymmetrically-distributed hydrophobic residues correspond to conserved positions in the logo shown in (A). Directions (arrows) and magnitudes of hydrophobic moments are indicated. Sequences begin with the top inner residue shown and extend clockwise, N- to C- terminal, into the page. Examples include the following taxa, ORFs, residues and sequences: (B) *Mmm *SC PG1; gi|42561534|ref|NP_975985.1| (LppQ); 243..278 WKTANVKTMRSMFSDTKQFNQDISSWNVSNVKNMKN. (C) *Mcc *Kid; gi|83319816|ref|YP_424254.1| (MCAP_0268); 210..245 WDTSNLETIDQMFVGAKKFNQDISKWDVSNVRIMDS. (D) *Mesoplasma florum *L1; gi|50365263|ref|YP_053688.1|; 492..527 WDTSKVTDMSNMFSGSSAFNGDISKWNTSSVTNMSG. (E) *Helicobacter hepaticus *ATCC 51449; gi|32265549|ref|NP_859581.1|; 638..673 KAKKFNQPLESWNVSNVANMRNMFGETDVFNQPLDK. (F) *Microscilla marina *ATCC 23134; gi|124008438|ref|ZP_01693132.1|; 243..278 NMGAMFSAAVAFNQPLEGWNTSQVTNMGGMFHWAKV. (G) *Salinibacter ruber *DSM13855 (plasmid pSR35); gi|83816872|ref|YP_446962.1|; 357..392. WDVSGVTDMSEMFEGAASFNQDISGWDVSNVTDMFE. (H) *Ostreococcus tauri*; gi|116055666|emb|CAL57751.1|; 852..887 NATEFNQDIAAWNTTSVANMAEMFSNAAAFNQNISA. (I) *Micromonas sp.* RCC299; gi|226517946|gb|ACO63939.1|; 509..544 WDTSSVTTMYRMFNEAAAFNQDIGRWDTSSVTDMKE.

First, a protein sequence logo (Figure 2A) representing the sum of individual motif sequences incorporated into the HMM shows that highly conserved residues occur periodically at distinctive positions in the canonical 25-residue repeat, whereas intervening residues vary greatly, as reflected by their markedly lower relative frequencies of occurrence at the respective positions of the HMM. These probabilities alone argue for the structural or functional importance of the conserved residues [5,34]. Notably, they correspond to amino acids of greater hydrophobicity, thereby forming a regular pattern of hydrophobic residues extending throughout tandem arrays of the motif (Figure 2A). Sequence-based predictions of secondary structure revealed recurring regions of helicity in tracts of the repeating sequence from diverse sources (data not shown), a feature also described in the prototype LppQ and LppC proteins of *Mmm *SC [15,16]. Most striking, however, were helical wheel projections of several representative tracts (Figure 2B-H) that revealed the strongly amphipathic character of these sequences, resulting from the asymmetric recurrence of conserved hydrophobic residues on one side of a helix. Amino acids at the variable positions, interspersed among the hydrophobic residues (Figure 2A), were more generally hydrophilic, consistent with their predicted interactions with polar environments. These characteristic amphipathic helical projections were common among 25-residue repeat units, they could be demonstrated using different phases of a repeated sequence, and they extended over the boundaries of adjacent repeats (Figure 2B-H). The strong amphipathic character associated with tracts of canonical domains was also confirmed by hydrophobic cluster analysis [35] of representative ORFs. Occasionally, hydrophobic residues at non-conserved positions affected the degree of amphipathicity. Structure-disrupting proline residues also occurred in some tracts of the motif, yet were often accommodated in amphipathic helical projections (Figure 2E). Not surprisingly, however, tracts with disrupted repeat patterns (for example, with contiguous non-canonical domains, or with gaps between canonical domains) also displayed corresponding interruptions in this secondary structure prediction (data not shown).

In the absence of experimental confirmation or *bone fide *models of a 3 D structure for any protein containing this HMM repeat motif, we speculate that the tandemly repeated domains may confer characteristics common to analogous helical repeat proteins with similar sequence features. Such proteins can display striking, extended architectures, resulting from the interactions of independently-acting domains forming modular scaffolded structures [2,5,36,37]. The strongly conserved amphipathic character of the HMM repeat motif described here is consistent with a comparable role that could mediate intra- or inter-molecular interactions, either with partnering domains or through the formation of complexes with other ligands [2,38,39]. Because increased numbers of tandem domains can dramatically augment structural or functional complexity by expanding the folding pathways available in repeat proteins [36,37], the large numbers of repeats in many HMM motif-containing ORFs could offer extensive ensembles of interacting domains. We observed that HMM-defined domains did not encode trans-membrane (TM) segments, nor did we find evidence for in-plane membrane anchor motifs [40] or other targeting signals in several examples analyzed. While we surmise that the domains do not independently mediate membrane interactions, their contribution to membrane-associated configurations, such as multi-domain trans-membrane channels, cannot be formally ruled out. Notwithstanding the need to resolve structures of the repeating domain in order to explore these possibilities, one clear hallmark predicted from sequence analysis is the strong amphipathic character of these regions. Importantly, this structured characteristic underscores their fundamental difference from a separate class of repeat proteins in which short, tandemly arrayed sequences predict intrinsically unstructured products [41,42]. It is noteworthy that such unstructured repeats occur in some families of surface proteins expressed in mycoplasmal taxa that also harbor ORFs with the HMM-defined repeat we report here [43].

A second hallmark of the HMM-defined domains is their high degree of protein sequence diversity, manifest in the hydrophilic amino acids interspersed between conserved hydrophobic residues (Figure 2A). Comparison of each individual domain sequence retrieved from the nr protein sequence database with all others retrieved from that database revealed extensive sequence diversity; 76.7% (2935 of 3825 total domains) had unique sequences. Sequence variation among domains within single ORFs was also striking, with the great majority representing tracts in which each domain represents a unique sequence (illustrated in Figure 1A). While the consequences of this sequence variability are unknown, it could reflect functional attributes of corresponding translation products, such as the diversity of ligand binding demonstrated in some repeat proteins with analogous characteristics [5,34]. The conserved secondary structure of HMM-defined motifs suggests an evolutionary history that preserved this common aspect, whereas diversification of more polar residues could represent the selection of distinct (unknown) advantages, or the mere accrual of "neutral" mutations that retain the structural integrity of domains. In either case, their combined sequence variability and structural modularity offers a highly adaptive conjectural framework for the acquisition of multiple biological roles.

**Figure 3:**
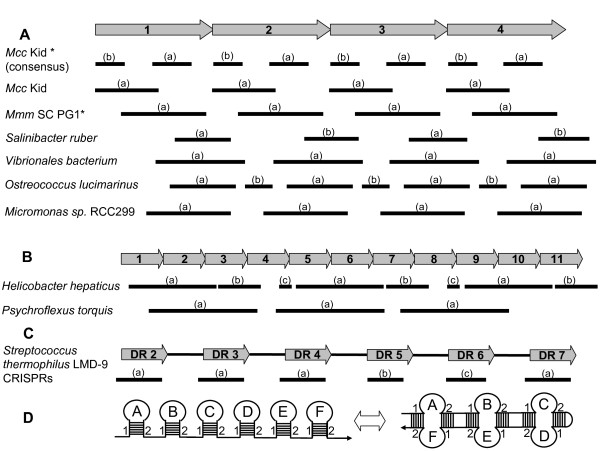
**Dyad symmetry in repeat-coding (PARCEL) sequences**. (A-B) Arrows depict tandem copies (1,2,3,...) of the canonical 25-amino acid (75-nucleotide) repeat representing the HMM logo in and Figure 2A. Bars below the arrows show locations and lengths of repeating palindromic motifs in relation to the HMM repeat-coding sequence. ORFs from the sources indicated at the left were used to derive the palindromic motifs shown, as described in Methods; these ORFs and features of the palidromes are detailed in Table 1 and Additional file 6. Lower case letters (a,b,c) indicate the single or multiple palindromic motifs derived from a particular source. Examples in (A) show repeating palindromic motifs with periodicities corresponding to the repeating HMM domains; those in (B) show longer palindromes spanning blocks of multiple HMM domains. The examples shown represent palindromic motifs derived from the sequences of individual ORFs, except in two cases indicated by asterisks (*): *Mcc *Kid (consensus) indicates palindromes generated from the composite of all HMM motif-containing ORFs in that genome; *Mmm *SC PG1 represents the palindrome derived from the composite sequences of two LPs shown previously to be expressed, LppQ and LppC. (C) The locations and lengths of palindromic motifs derived in the same manner from a region of CRISPR repeats are shown, corresponding to known palindromic DR repeats in the sequence (arrows), separated by spacer regions. (D) Cartoon illustrates the concept that multiple tandem palindromes of like sequence (stem-loops A through F) can form extended and diverse palindromic structures.

In addition to these striking features of the repeating protein sequences, we also found that the underlying sequences encoding these HMM-defined domains consistently displayed repeating elements with dyad symmetry. Searches for statistically-defined palindromic motifs [44] in DNA sequences encoding tracts of the domains revealed a variety of such regions, corresponding to the tandemly repeated canonical protein sequence of the HMM (Figure 3; Table 1). This feature was present in ORFs from diverse phylogenetic groups and environmental niches. The exact palindromic motif calculated for a particular ORF, or from datasets representing multiple ORFs, varied in sequence, length and phasing, relative to the repeating protein domain (Figure 3; Table 1, Additional file 6). Some palindromic regions recurred with the same period as that of the HMM (Figure 3A), whereas others spanned repeating block patterns corresponding to multiple HMM domains (Figure 3B). To verify these statistically derived patterns, multiple randomizations of input DNA sequences were shown to completely ablate the palindromic motifs, thereby ruling out a random occurrence in these coding sequences (data not shown). As a frame of reference using known repeats with dyad symmetry, we applied the same methods to derive motifs from well-characterized CRISPR repeat sequences [45-47]. As expected, the resulting motifs corresponded in location and sequence to the respective palindromic DR repeats in the CRISPR regions examined (Figure 3C; Additional file 6 sheet 2). The palindromic motifs derived from repeat sequences encoding HMM domains (and the actual sequences from which they were statistically generated) represented imperfect dyad symmetries, as do many CRISPR repeats [45,46,48]. Furthermore, as anticipated, they matched the corresponding authentic sequences encoding HMM domains with a range of scores (E-values) analogous to those obtained by matching CRISPR-derived palindromic motifs with their corresponding authentic sequences (see Additional file 6).

**Table 1:** Representative palindromic motifs associated with PARCEL coding regions

Sources and features of input sequences analyzed			Features of derived palindromic motifs		
**Taxon**	**PARCEL-containing ORFs analyzed (Locus_tag)**	**No. of HMM domains in ORFs**	**Palindrome motif (MEME)**	**Motif sequence** **(Symmetrical residues underlined)**	**Length (nt)**

*Mcc *Kid (consensus)	Sum of 42 ORFs listed in Additional file 7	226	(a) (b)	GTTGGTTGGAGCTGCCAGCTCCAACCAAC TGAGACACTTCAAGTGTCTCA	29 21

*Mmm *SC PG1	MSC_1021 (LppQ) plus MSC_1005 (LppC)	14	(a)	GGAAAAAGCATGGTTAAAATGTTTTGCGCCGCAAAACATTTTAACCAAGCTTTTTCC	57

*Mcc *Kid	MCAP_0311 (in Tra I)	10	(a)	TGAAACACTCCGCCCGTAACCGATACGGGCGGAGTGTTTCA	41

*Salinibacter ruber*	SRU_p0003	12	(a) (b)	GAAACCTTCAACCAACATATATCTTGGTTGAACGTTTC TACAATCGAAATATCCCAACTTCGGATATTTCGATTGAA	38 39

*Vibrionales bacterium *SWAT-3	VSWAT3_25559	16	(a)	TGACCGTAGGCGTATGCCATTCACCAATCGATTGGTGAAAGGCATACGCCTACGCACA	58

*Ostreococcus lucimarinus*	OSTLU_3281	9	(a) (b)	CCACGAGCTTCAACTTCGACATGTCGAACTTGAACCTCGACG GAACATGTCAGACATGTTC	42 19

*Micromonas sp*. RCC299	MICPUN_102633	9	(a)	CAAATTATCCCAAAAGCCGATGCGTTCAATCAACCCATCGGCTTTTCGGAAAATTTG	57

*Helicobacter hepaticus*	HH0050	19	(a) (b) (c)	CTAACGTTGGAAACATGCCAGCTATCTAAGGGGTGGCTCAGGTATTTAGCCAGCCCAAACATAACACGCATACAAGCAACGTTGCAAGTATCCCAGAAATCTATGGGCAGGCTAAAAACCTCAGCCACCCCAAACAAACCTGGCATGTATCCAACGTTAG CATGTGTGGAACGTTGCTTGTGTCCCATTTATCTAACCGCCCGTTAGATAAATGGGACACAAGCAACGTACCACACATG CCAGCCTTCAGAAGGCTGG	160 79 19

*Psychroflexus torquis*	P700755_04362	17	(a)	CTTACAACCCACCCCTCGAGGGGTGGGATGAAAGAAGTGGCACTAATAAGCATGGCATGCATATTAGTGCCACTACTTACAACCCACCTCTTAAGAGGTGGGATGAAAGAAGTGGCACTAATAAGCATGCCATGCATATTAGTGCCACTACTTACAACCCACCCCTCGAGGGGTGGGATGAAAG	184

DNA sequences of PARCEL-containing ORFs from several representative sources were analyzed for the occurrence of palindromic motifs as described in Methods. The derived sequences and features of palindrome motifs that correspond to the canonical HMM-defined repeat pattern are described. Additional details are shown in Additional file 6.

The overall significance of the palindromy in the coding sequences of HMM-defined domains, whether in extant taxa or in their evolutionary antecedents, is not clear. Interestingly, because tandemly repeated palindromes of like sequence can in principle form extended palindromic regions (Figure 3D), long tracts of these repeats may assume numerous configurations, potentially endowing single strand regions of DNA or RNA with properties that could influence gene expression, mobilization or other interactions with host genomes that have been implicated both for non-coding [45,46,48] and protein-coding [49-54] palindromic elements. However, mutational diversification of these protein coding sequences over time may have diminished more pronounced dyad symmetry in ancestral precursors, particularly if palindromy *per se *was not a selected trait. Moreover, while domains with distinctive protein sequences (Additional file 1, sheet 2) or codon usage patters (see final section of Results and Discussion) appear to dominate some lineages, meaningful comparison and interpretation of the underlying DNA sequences may be challenging, due to differences in the genomic context (e.g., G+C content and codon usage biases affecting wobble positions) and the multiple mechanisms available for propagation of particular sets of coding regions (e.g., reiterations within in tandem arrays vs. acquisition, duplication or recombination of ORFs.) Regardless of its ultimate source or function, the underlying dyad symmetry associated with coding regions of the HMM protein domain is a central aspect of this repeating pattern, and is incorporated into the PARCEL descriptor that we employ hereafter.

### PARCEL domains add diversity to membrane proteins

The expression of PARCEL-containing proteins was first established for LppQ and LppC [15,16], two surface LPs from a single mycoplasmal taxon represented by the type strain *Mmm *SC PG1 [RefSeq: NC_005364] [55], of the *M. mycoides *cluster. We therefore exploited recent genomic sequences and proteomic data from a second species of this group, *M. capricolum *subsp. *capricolum *strain Kid (*Mcc *Kid) [RefSeq: NC_007633], in order to evaluate the role of PARCELs in diversifying the surface protein repertoires in this group of animal pathogens. These two taxa (i) are very close phylogenetic relatives [17,18] with genomes showing marked synteny and gene orthology [56], (ii) include the greatest number and diversity of PARCEL-containing ORFs in any single taxon (*Mcc *Kid; see Additional file 7) and (iii) express additional families of phase-variable repeat proteins that are structurally and antigenically distinct [43,57,58]. We earlier established a partial "membrane proteome" of *Mcc *Kid using nanoflow capillary LC-MS/MS to identify and map tryptic peptides (generated from a membrane protein-enriched fraction of the organism) to their corresponding genomic sequences [43]. Extending preliminary observations reported from those studies, we confirm here that at least four PARCEL-containing LPs are expressed by this organism: MCAP_0268, MCAP_0704, MCAP_0720, and MCAP_0721 (denoted in Additional file 7). Peptides corresponding to unique sequences throughout these ORFs, including PARCEL domains, verified the expression of full-length translation products (Additional file 8). Consistent with their presence in detergent-phase preparations, each product was encoded as a pre-protein containing a lipobox that predicts LP processing [59], with the mature protein ultimately anchored in the single plasma membrane. Consequently we compared these expressed products of *Mcc *Kid with LppQ and LppC of *Mmm *SC PG1, in order to document possible variation between the two taxa manifest in these six surface proteins.

BLASTp comparisons (of regions outside PARCEL tracts and cleaved SP regions) indicated that MCAP_0268 and MCAP_0704 of *Mcc *Kid were distinct from one another, and had no counterparts in *Mmm *SC PG1. Similarly, LppQ (MSC_1021) and LppC (MSC_1005) [55] were each distinct and were selectively present in *Mmm *SC PG1. Contrasting these unique LPs were the expressed LPs MCAP_0720 and MCAP_0721. These represented adjacent in-paralogs with high sequence similarity, which was also shared by one predicted PARCEL-containing ORF (MSC_0773) in *Mmm *SC PG1 [55]. In this context, as modules embedded in orthologous or non-orthologous framework sequences, PARCELs contributed directly to the sequence variability of expressed surface proteins. Notably, each of the 42 individual PARCEL domains represented in these six verified translation products had a unique sequence (data not shown).

These findings formally demonstrate expression of a new family of surface LPs representing taxon-specific variants that distinguish members of the *M. mycoides *cluster. Although the full ramifications of PARCEL domain repeats in these proteins are not determined, LppQ and LppC are known to be prominent surface antigens of *Mmm *SC. Interestingly, in both cases the dominant B cell epitopes recognized in the natural host reside outside tracts of PARCEL motifs [15,16]. We speculate that the domain architecture could affect either the proper recognition, or immunogenicity, of these repeat regions. Regarding their role as surface proteins, it is noteworthy that some PARCEL-containing ORFs in *Mcc *Kid (denoted in Additional file 7) display adjunct features shared by other phase-variable membrane protein families expressed from this genome [43] and the genome of *Mmm *SC PG1 [55,57,58]. Specifically, contingency loci comprising homopolymeric or dinucleotide VNTRs reside in the 5' flanking regions of some PARCEL-containing ORFs encoding LPs (including the LP expressed from MCAP_0268) and other membrane proteins. The occurrence of PARCEL sequences in genes encoding phase-variable surface membrane proteins, governed by modular contingency loci, may reflect promiscuous co-opting of the domain for adaptive variation by these organisms.

**Figure 4:**
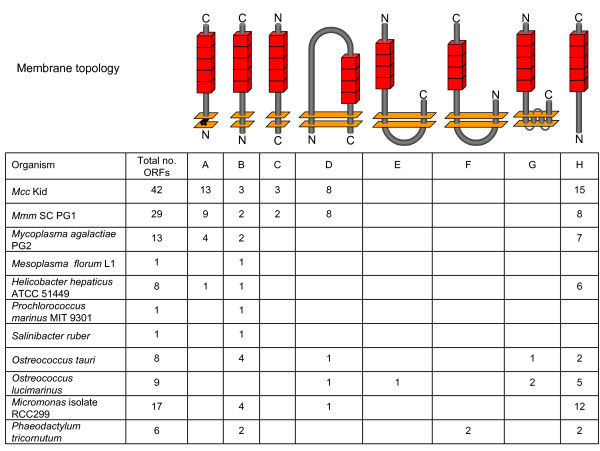
**Representative genomic inventories and predicted topologies of membrane proteins containing PARCEL domains**. Inventories of PARCEL-containing ORFs in the genomes of taxa listed at the left are shown. The total ORFs per genome, and the number representing various categories of membrane protein (A-G) or lacking membrane-targeting sequences (H) are indicated. Cartoons show the locations of PARCEL domain tracts (tandemly arrayed red boxes) relative to the N- and C-termini of the respective ORFs (rods), and to the plasma membrane (bilayers represented by parallel planes). Topologies include: (A) monotopic bacterial LP, anchored by N-terminal lipid modification; (B and C) bitopic TM proteins of different orientations; (D-G) polytopic proteins with two (D-F) or more (G) TM segments. Topology (B) includes proteins with predicted SPs (other than LPs). Topology (G) represents proteins with any number (> 2) of TM domains. Locations of PARCEL domains in ORFs are not to scale and depict only their positions relative to the features shown. The inside or outside of the membrane is not indicated although PARCEL domains were most often predicted to lie outside.

A very recent study of transposon insertional mutants of *M. agalactiae *[60] has implicated the products of two PARCEL-containing ORFs (among diverse other ORFs) in adaptation of that organisms to growth in cell culture [60]. Our manual curation of those ORFs (MAG1330 and MAG3260) [RefSeq: NC_009497] suggests that they encode LPs containing a homopolymeric (poly G) VNTR tract in the N-terminal coding region that could be subject to phase variable expression through frameshift mutation. A second study by this group [61] directly documents the expression of two other PARCEL-containing LPs in the same organism (MAG64080/MAG6490 and MAG2430). These and our studies collectively offer evidence that PARCEL ORFs contribute to the adaptive strategies of the two mycoplasmal lineages harboring significant genomic repertoires of these ORFs. While an adaptive role has been previously inferred for PARCEL-containing ORFs based on these large repertoires *per se *[19], we caution against this general notion by comparing *Mesoplasma florum*, a plant pathogen and the closest ancestral relative of the *M. mycoides *cluster having a complete genome sequence (Additional file 4B). This organism harbors only one PARCEL-containing ORF, encoding a bitopic membrane protein (Figure 4, category B). Offering only a limited potential for diversification, this example highlights the possibility that more specific roles are associated with these repeat domains in some types of membrane proteins.

To examine more generally the spectrum of PARCEL-containing ORFs with membrane-targeting sequences, we surveyed a selection of taxa having fully sequenced genomes. Figure 4 illustrates representative inventories from diverse organisms interrogated with our HMM, showing that repeats occur in several categories of integral membrane proteins with monotopic or various multitopic configurations. These ORFs were broadly categorized by signature sequences marking bacterial LPs (characterized by lipobox processing motifs in their SPs [59,59], or TM sequences (found in all taxa, and including other SPs that effect membrane translocation regardless of subsequent cleavage [62,63]). In all such ORFs examined, PARCEL domains resided on one side of the membrane. Most were predicted to lie on the external face, but are not depicted as such due to the known ambiguity of sequence-based predictive algorithms [62]. Individual genomes varied greatly in the number and types of PARCEL-containing ORFs present (Figure 4), ranging from one ORF in many taxa, to a multitude in others such as *Mcc *Kid and *Mmm *SC PG1, ironically among the smallest. Some genomes encoded multiple types of membrane proteins carrying the motifs. Even the single, or few PARCEL-containing ORFs in some genomes displayed significant sequence variation among individual domains (data not shown). Overall, the variety of encoded membrane proteins harboring PARCEL sequences argues for a prominent and varied role in this context, possibly reflecting processes specific to particular organisms. One noteworthy group of these ORFs lacked membrane targeting sequences, however (Figure 4, category H). These typically occurred in genomes along with ORFs having such signals. Whether these "untargeted" ORFs represent pseudogenes, encoded translation products destined for cytoplasmic compartments, or truncated artifacts from automated annotation is not known.

### PARCELs are subject to HGT and intra-genomic mobilization

The variable sequences and numbers of repeats in PARCEL-containing ORFs hampers analysis of HGT using orthology to compare their phylogenetic congruence with the organisms harboring them [64]. Nevertheless, by comparing the genome sequences of related organisms within assorted phylogenetic clades, we discovered markedly different repertoires and intra-genomic distributions of PARCEL-containing ORFs. Moreover, in some genomes these ORFs were exclusively associated with mobility elements. The following examples from eucaryotes and bacteria illustrate these features, which support the notion that PARCEL sequences are in flux, both among and within genomes.

The chromosomes and organelles of all known eucaryotic taxa containing PARCEL sequences (Additional file 4A) have been fully sequenced and the genomes extensively compared, revealing for each taxon a history of exogenous gene acquisition that has driven speciation and diversification [65-69]. Genome sequences from the Stramenopiles include two diatoms, *Phaeodactylum tricornutum *[67] and *Thalassiosira pseudonana *[69]. We identified six PARCEL-containing ORFs in *P. tricornutum*, each on a different chromosome, and none in *T. pseudonana*. The former belongs to a more recently-diverged group (pennates), thought to be extensively diversified by the acquisition of exogenous genes, and by rearrangements mediated by the retrotransposon elements prevalent in the genome [67]. This study applied orthology searches that identified hundreds of genes in each genome belonging to "bacterial" lineages, prompting speculation that massive and successive uptakes of bacterial genes occurred very early, before their divergence (bacterial genes shared by these taxa) and subsequently by each taxon (bacterial genes unique to one or the other). Parenthetically, only one PARCEL-containing ORF [GenBank: EEC50352.1] was listed in the inventory of "bacterial" genes of *P. tricornutum *[67], possibly underscoring the shortcomings of orthology in the classification of PARCELs. Overall, this macro-scale comparison shows a striking difference in PARCEL content, and a distribution of these ORFs consistent with their selective acquisition, subsequent mobilization and propagation within a genome. That chloroplast or mitchondrial genomes of these organisms [67] lacked PARCELs offers no support for the direct transfer between organelle and host chromosomes, but is consistent with an exogenous source for these sequences.

A second eucaryotic clade, Mamiellales, includes two closely related species, *Ostreococcus tauri and O. lucimarinus *(Additional file 4A), reflecting early speciation events in the green algal lineage [65,66]. Of 20 chromosomes in *O. tauri *(comprising the smallest known eucaryotic genome) and 21 in *O. lucimarinus*, 18 show strong synteny between the species and consequently define pairs (Table 2). Additional unpaired chromosomes in each organism are thought to be acquired exogenously, as is one highly rearranged and distinctive chromosome (Chx 2), present in both [66]. Several PARCEL-containing ORFs of various predicted membrane topologies occurred in both genomes (Figure 4) but were distributed in very distinctive patterns among the respective chromosomes (Table 2), showing (i) the selective presence of some ORFs on a paired chromosome of one species versus the other and (ii) the presence of ORFs on all chromosomes predicted to be of exogenous origin. These features support the notion that PARCEL-containing ORFs were mobilized, and prompt speculation that they were introduced during the predicted HGT events that shape these genomes. Further dispersal in the genome could have ensued, perhaps in part through the action of transposon elements that abound also in these taxa [65,66]. Again, no PARCEL sequence was found in the mitochondrial or chloroplast genomes of these organisms, nor in the recently-reported genome of one virus known to infect *O. tauri *[70]. Finally, the genome sequences of two related taxa from a separate branch of Mamiellales, *Micromonas *isolates RCC299 and CCMP1545 (Additional file 4A), have recently been reported [68]. We identified 17 PARCEL-containing ORFs in the former, distributed on 6 different chromosomes, and 8 such ORFs in the latter (not assigned to chromosomes). These taxa share only 90% of their genes and are considered more divergent than ostreococcal species, as a consequence of gene acquisitions that selectively expanded unique repertoires in each organism [68].

**Table 2:** Distribution of PARCEL-containing ORFs among the chromosomes of two *Ostreococcus *species

Chromosome designation [number of PARCEL-containing ORFs]*		comments
*O. lucimarinus*	*O. tauri*	

**chx. 1 [1]**	chx. 1	

**chx. 2 [1]**	**chx. 2 [1]**	Chromosome 2 of each species is potentially of exogenous origin*

chx. 3	chx. 3	

**chx. 4 [1]**	chx. 4	

chx. 5	chx. 5	

chx. 6	chx. 6	

chx. 7	chx. 7	

chx. 8	**chx. 8 [3]**	

**chx. 9 [1]**	chx. 9	

chx. 10	chx. 10	

chx. 11	**chx. 11 [1]**	

chx. 12	chx. 12	

chx. 13	chx. 13	Chromosome 13 of *O. tauri *shares regions with two chromosomes of O. lucimarinus*

**chx. 14 [2]**	chx. 15	

chx. 15	chx. 16	

chx. 16	**chx. 17 [1]**	

chx. 17	chx. 18	

**chx. 18 [1]**	------------	Chromosome 18 of *O. lucimarinus *is potentially of exogenous origin*

------------	**chx. 19 [2]**	Chromosome 19 of *O. tauri *is potentially of exogenous origin*

**chx. 19 [1]**	chx. 20	

**chx. 20 [1]**	chx. 14	

chx. 21	chx. 13	Chromosome 13 of *O. tauri *shares regions with two chromosomes of O. lucimarinus*

* Equivalent paired chromosomes are listed on the same line. Those harboring PARCEL-containing ORFs are indicated in bold font with the number of ORFs in brackets. Chromosomal designations and interpretations are described in [66].

In bacteria, the completed and fully assembled genome sequences from multiple taxa revealed a selective distribution of PARCELs among the members of particular lineages, for example among subgrouops of Firmicutes, Cyanobacteria and Tenericutes (Additional files 3, 4B and 5). A striking example is found (Additional file 5) in the genomes of a tight cluster representing the cyanobacterium *Prochlorococcus marinus*. Of twelve fully sequenced isolates, only two (MIT 9215 and MIT 9301) contained PARCEL sequences, with each harboring a single PARCEL-containing ORF encoding a bitopic TM protein (Figure 4, category B). These ORFs have significant sequence similarity and comparable organization of PARCEL tracts. Modest sequence differences within and outside PARCEL domains reveal only limited divergence. However, each ORF resides in a completely different genomic location, devoid of apparent mobility genes. These findings are most consistent with the selective acquisition of a PARCEL-containing ORF by these taxa or their close ancestor, and/or further mobilization in the genome. The alternative scenario, involving the selective loss of common ancestral orthologs by the ten other isolates, cannot be formally ruled out but is considerably less likely.

That PARCEL-containing ORFs can be mobilized and are subject to HGT was further evidenced in the complete genome sequences of some bacteria where they reside exclusively within mobility elements. In one case, the single such ORF in the genome of *Salinibacter ruber *[71] occurs on the unique plasmid of that organism, pSR35 (35,505 bp) [RefSeq: NC_007678], along with 31 other ORFs. This ORF (SRU_p0003) [RefSeq: YP_446962.1] encodes a bitopic TM protein (Figure 4, category B) with a possible SP sequence. It contains a tract of 12 PARCEL domains predicted to lie external to the plasma membrane. The sequence of each domain is unique, compared to others within this ORF and to all others in the nr database (determined by BLAST). The plasmid encodes a transposase of the IS*5 *type, different from the IS*1 *type encoded on the single *S. ruber *chromosome. Parenthetically, this environmental hyperhalophile is reported to exchange genetic information with haloarchaea that share its extreme habitat [71]; to date no available archaeal genome sequence has revealed PARCEL domains (Additional file 5). In a second case, the single PARCEL-containing ORF in the genome of the soil bacterium *Arthrobacter chlorophenolicus *A6, resides on the larger of two plasmids in that organism, pACHL01 (426,858 bp) [RefSeq: NC_011879] along with 553 other ORFs. This ORF (Achl_4487) [RefSeq:YP_002478255.1] predicts a TM protein (possibly with SP sequence) of similar topology to that found in *S. ruber*. It contains a tract of 4 PARCEL domains, each again having a unique sequence. Together these examples (i) confirm that PARCEL motifs of two environmental bacteria from disparate lineages and habitats are exclusively associated with distinctive extrachromosomal replicons and (ii) directly identify vehicles that could mobilize specific PARCEL sequences.

**Figure 5:**
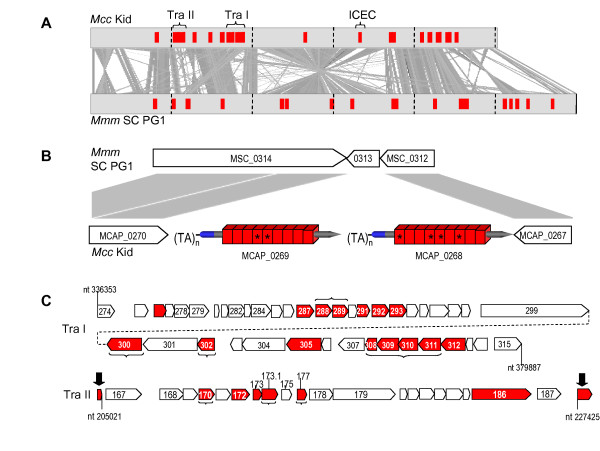
**Distribution and features of PARCELs in genomes of *Mcc *Kid and *Mmm *SC PG1**. (A) Linearly-aligned chromosomes of *Mcc *Kid and *Mmm *SC PG1 are compared using BLASTn and ACT to visualize regions of high sequence similarity and co-linearity (shaded). Locations of PARCEL-containing ORFs are marked in red; clusters of ORFs appear as wider blocks. Integrative and conjugative elements (Tra I and Tra II islands; ICEC) are bracketed. (B) Representative configuration of PARCEL-containing ORFs in *Mcc *Kid. ORFs encoding the expressed LP MCAP_0268 and predicted LP MCAP_0269 are flanked by conserved regions corresponding to an "empty site" in the chromosome of *Mmm *SC PG1. PARCEL-containing ORFs (rods) together contain 18 domains (boxes), each having a unique sequence. Non-canonical domains are marked (*). SPs containing bacterial lipoboxes are shaded blue. VNTR dinucleotide (TA) tracts 5' of each gene are indicated. (C) Organization of PARCEL-containing ORFs in the Tra I and Tra II genomic islands of *Mcc *Kid. Ends of each element are indicated by nucleotide numbers [RefSeq: NC_007633]. Arrows show ORFs within Tra I and Tra II and the predicted direction of transcription. PARCEL-containing ORFs (shaded red) are described further in Additional file 7. Numbers within or above some arrows denote reference locus tags. Brackets indicate PARCEL-containing ORFs lacking membrane targeting signals. Vertical black arrows indicate the N- and C-terminal portions of a PARCEL-containing ORF (encoding a membrane protein) that is interrupted by insertion of Tra II.

Comparison of the closely-related genomes of *Mcc *Kid and *Mmm *SC PG1 also revealed a subset of PARCEL-containing ORFs selectively associated with large mobility elements. These genomes harbor a total of 42 and 29 PARCEL-containing ORFs, respectively (Figure 4; Additional file 7), distributed throughout their chromosomes (Figure 5A). Many of these ORFs reside at the same locus in each genome; others occur selectively in one or the other, as single ORFs or tandemly organized paralogs occupying sites that are "empty" in the opposite genome (Figure 5B). Typically these sites are in regions of housekeeping genes, or near IS elements that extensively populate the *Mmm *SC PG1 genome [55]. A striking exception to this pattern is the clustering of several PARCEL-containing ORFs within two large islands in the *Mcc *Kid genome, Tra I and Tra II (Figure 5C). Described in part previously [72] and annotated in the genome sequence [73], these islands represent a newly-recognized class of element, containing ORFs that resemble mobility genes that are clearly different from those identified in other integrative and conjugative elements (ICEs) [74] annotated and described in *Mcc *Kid [19,73] and in other mycoplasmas [75-77]. Tra I and Tra II harbor 15 and 5 PARCEL-containing ORFs, respectively (Additional file 7), together accounting for the larger inventory of these ORFs in the *Mcc *Kid genome over that of *Mmm *SC (see also Figure 5). One intriguing aspect of the PARCEL-containing ORFs associated with these Tra elements is the marked absence of membrane-targeting signatures from many (Figure 5C), a feature (confirmed by manual curation) that distinguishes them from most others in the genome (Additional file 7). Moreover, some of these ORFs (particularly in Tra I) are organized in tandem, have similar orientations, and share a characteristic sequence motif outside tracts of PARCEL domains that further sets them apart (motif is described in Additional file 7). The unique configuration and character of these ORFs support speculation that at least some were acquired with the elements. Interestingly, individual PARCEL domains in these "untargeted" ORFs display significant sequence diversity. Whether or not they are transcribed or translated, these ORFs are reservoirs containing variant PARCEL coding sequences that could be exploited through recombination with other expressed ORFs in the genome.

No Tra island or ICE in mycoplasmas has been directly shown to mediate conjugative transfer, however some (including Tra II) are known to exist in extra-chromosomal forms and to integrate at multiple chromosomal sites [72,75,76]. Overall, these findings strongly implicate these large elements in the acquisition and mobilization of PARCEL-containing ORFs in the *M. mycoides *cluster. A recent comparison of genomes from two strains of *M. agalactiae *[61] has revealed a dynamic role of analogous large mobile elements in shuffling PARCEL-containing ORFs in that species. Events mediating the introduction, duplication, loss or reshuffling of PARCEL-containing ORFs in these genomes appears to be complex. In the two members of the *M. mycoides *cluster examined, for example, no evidence of Tra II, and only a remnant of Tra I, was found in the *Mmm *SC PG1 genome (Figure 5A). In addition, the boundaries of Tra II indicate its precise insertion into a pre-existing PARCEL-containing ORF encoding a TM protein, now annotated as MCAP_0166 and MCAP_0188, corresponding to the disrupted N- and C-terminal portions of the gene, respectively (Figure 5C; Additional file 7). Multiple incursions of these elements have clearly shaped the PARCEL content of genomes during the divergence of subspecies within the *M. mycoides *cluster. On the other hand, some PARCEL-containing ORFs may pre-date this repertoire expansion, and their exchanges among taxa of this group, as evidenced by their presence also in *M. florum*. This is a commensal mycoplasma of the same phylogenetic heritage (Additional file 4B), which is found on lemon tree flowers, a completely separate environmental compartment.

### PARCELs are exogenously derived from the mobilome

The apparently random distribution of PARCELs among disparate phylogenetic groups and environments is most easily reconciled with transmission by various forms of HGT, now known to occur among unicellular organisms from all three domains of life [27,67,78-80], While no single origin, nor direct evidence for the transfer between diverse phylogenetic clades can be demonstrated for PARCEL-containing ORFs, their selective association with distinctive mobility elements in particular clades argues that subsets were likely introduced and fixed into lineages, due in part to the restricted host ranges of most vehicles. This notion is further supported by the independent observation of clade-specific features that are affiliated with some groups of PARCELs. Most notably, *Mollicutes *utilize UGA as a Trp codon rather than a translation termination signal and show a strong bias for this codon over the alternative UGG (Trp) [81]. We confirmed this extreme bias in PARCEL sequences of *Mollicutes*, wherein UGA nearly always encodes the highly conserved Trp residue (Figure 2) in the first position of the motif (data not shown). This implies that an ancient subset of PARCELs was irreversibly fixed in *Mollicutes *(Additional file 4B), concurrent with the evolution of its distinctive codon usage. As a corollary, this also argues indirectly that the conserved Trp residue and associated domain structure may have been a selected trait of PARCEL sequences during their acquisition and exploitation in particular clades of *Mollicutes*. From a genome-centric perspective, the ultimate exogenous source of PARCEL sequences is elusive. They are perhaps best viewed as components of the vast array of mobile genetic information that is conveyed among select microbial communities by gene-centric mechanisms [78,82-84]. They clearly contribute to the "accessory gene" pools that distinguish individual taxa and contribute to the "pan-genomes" of related organisms [10,11]. PARCELs have markedly expanded the pan-genome of the *M. mycoides *cluster, with some genomes reflecting extensive colonization, despite their reduction in size [81].

Our study documents the widespread mobilization of ORFs containing tandem arrays of PARCEL domains, consistent with their ultimate dissemination by HGT and intra-genomic propagation. However, the palindromic nature of the DNA sequences encoding PARCEL domains is also noteworthy *per se*, in regard to their possible origins, propagation, and properties in tandem arrays. Of the many palindromic sequences found among procaryotic genomes [85], some specialized subsets represent exogenous protein coding sequences integrated into assorted housekeeping genes of intracellular bacterial parasites including *Rickettsia *[51,54] and *Wolbachia *[50] as well as the archaeon *Methanocaldococcus jannaschii *[49]. These stand-alone, "selfish" coding elements appear to be in-frame insertions of several codons, introduced at assorted positions typically located in non-structured segments of globular proteins [51]. By extension of such findings, the insertion and subsequent propagation of palindromic coding sequences is proposed to be an important mechanism for *de novo *evolution and diversification of proteins [52,53]. The dyad symmetry of PARCEL coding sequences could reflect an ancient property (possibly diminished over time by sequence divergence) that was instrumental in their original introduction into assorted ORFs. An interesting feature predicted for progenitor palindromic coding sequences is the presence of overlapping ORFs on the opposite strand [52]; this characteristic is found in many (but not all) PARCEL coding regions (data not shown). The palindromic motifs detected in PARCEL coding sequences show considerable variation in predicted energies of stem-loop formation, consensus sequences and boundaries, resembling the analogous variations reported in subclasses of the elements referenced [49-51,54]. However, protein sequences in PARCEL tracts differ in several ways, including their tandem repetition, conserved amphipathic secondary structure, association with membrane proteins and occurrence in genes of unknown function. Hence these regions represent a newly-described class of palindromic coding sequence.

## Conclusions

We characterize a widely dispersed, versatile repeating protein domain and coding sequence, for which we recommend the moniker "PARCEL" (**P**alindromic **A**mphipathic **R**epeat **C**oding **EL**ement) to describe its distinguishing and generalized features. Because the HMM used to define this motif identifies regions generally corresponding to those recognized by HMMs DUF285 (IPR005046) and TIGR02167 (IPR011889), the attributes we ascribe also apply to those motifs. Tandem repeats of the protein motif are predicted to form modular domains with potentially diverse folding pathways, and display highly variable hydrophilic sequences. Both features provide a plasticity that could endow diverse biological functions in this newly-characterized class of repeat proteins and coding sequences. Our findings support the following scenarios to explain the dissemination and deployment of PARCELs among extant genomes: (i) PARCEL-containing ORFs have been stochastically distributed among phylogenetic groups by multiple vehicles, possibly as expressed ORFs or as coding reservoirs and (ii) they propagated and evolved within some genomes through further mobilization, rearrangement, gene duplication and decay, (iii) they are prevalent in membrane-targeted proteins and may provide selective advantages in that context, either through a conserved domain structure or their extensive sequence variability (possibly generated prior to acquisition or through ensuing mutation) and (iv) clade-specific subsets of the elements helped diversify the surface protein repertoire in specific lineages of at least one bacterial group (*Mollicutes*). The dynamic nature of PARCELs conforms most readily to the concept of a mobilome [78], a network of genomic nodes connected by mobilization of genetic information through multiple pathways within shared habitats. In this regard PARCELs are emblematic of repetitive protein motifs whose origins and distribution should be examined in the context of gene flux.

## Methods

### Hidden Markov model and interrogation of databases

The HMMER package [20] was used to construct the profile HMM and to search protein sequence databases. The HMM is provided in Additional file 9. Searches of the nr and env_nr databases were performed with an E-value ≤ 1. ORFs identified by the HMM were subsequently searched for COGs through the NCBI resource [24]. To inventory all ORFs in a representative taxon, the corresponding annotated genome sequence was individually searched with the HMM. A logo representing the HMM was generated as described in [86], using relative entropy to indicate the prevalence of amino acid residues at each position.

### Phylogenetic tree construction

A phylogenetic tree based on 16 S small-subunit rRNA gene sequences of bacterial taxa harbouring PARCEL-containing ORFs was constructed using resources available through the Ribosomal Database Project (rdp) [87]. Sequences were aligned using rdp's aligner [88,89] and a distance matrix was generated using the Jukes-Cantor corrected distance model [87]. The tree was created with rdp's Tree Builder, using Weighbor, a weighted version of Neighbor Joining [90]. The calculated trees were further refined using the program MEGA version 4.0 [91].

A phylogenetic tree based on the 18 S small-subunit rRNA gene sequences of eucaryotic taxa harboring PARCEL-containing ORFs, or representing other diverse groups, was generated using the program MEGA version 4.0 [91]. Sequences were aligned using ClustalW and the phylogenetic tree was subsequently constructed using the neighbor-joining method [92], Maximum Composite Likelihood [93] correction, and 1000 bootstraps.

A phylogenetic tree based on the 16 S small-subunit rRNA gene sequences of bacteria and archaea having fully sequenced and assembled genomes was generated using Infernal [94] to align sequences of each group and MUSCLE [95] to merge the two alignments to produce a combined alignment. This alignment was used to construct a maximum likelihood tree using RAxML [96].

### Sequence analyses

Nucleotide and protein sequence comparisons were performed, respectively, using BLASTn (without filter) and BLASTp software available through NCBI [24]. ORFs were examined and curated using Artemis version 7 [97]. Comparisons of genomes and regional sequences were made with BLASTn output files visualized in ACT version 4 [98] with a default setting of 100 nt as the minimum window for displayed matches. Protein sequences were analyzed with multiple tools available through Biology WorkBench [99], including secondary structure predictions using the PELE suite of programs, alignments using ClustalW, and membrane topologies using TMHMM2.0 (scoring TM regions with probabilities > 0.2). LP signal peptides were identified by lipobox search patterns described elsewhere [59] and available through InterPro [22]. Additional protein sequence-based predictions included helical wheel projections [100,101] using whole-residue interface hydrophobicity scales [102], hydrophobic cluster analysis [35] and in-plane membrane anchor analysis [40]. Other protein motifs and 3 D structural predictions associated with PARCEL- or DUF285-containing ORFs were evaluated through the InterPro resource [22]. A protein motif associated with subsets of PARCEL-containing ORFs associated with mobility elements was generated using MEME [44] and used to query ORFs by BLASTp. The uniqueness or identities of individual motif sequences in the nr or nr_env data sets were determined by comparing each sequence shown in Additional files 1 or 2 (sheets2) with all others in each dataset. Features of some genomes and ORFs were acquired through NCBI or the DOE Joint Genome Institute Integrated Microbial Genomes resource [103].

### Palindrome analysis

Palindromic motifs in DNA sequences were identified using MEME (version 3.5.7) [44], with default settings for palindromes only, and limits of 6 nt (minimum) and 300 nt (maximum) for the motifs recovered. Palidromic motifs < 10 nt in length were excluded as background. Input datasets for these analyses included the entire DNA sequences of individual ORFs or, where noted, a set of multiple ORFs. Multiple, randomly shuffled input DNA sequences were used as negative controls. CRISPR sequences used as positive controls were obtained and analyzed through the CRISPRdb database [47].

### Proteomics

The generation of tryptic fragments from detergent phase-fractionated proteins of *Mcc *Kid, LC-MS/MS techniques and mapping of tryptic peptides to ORFs encoded by the *Mcc *Kid genome sequence have been described in detail elsewhere [43]. In addition to SEQUEST [104] analysis (Thermo Finnigan; Bioworks v3.1), X!Tandem analysis was also performed [105], and peptide and protein probabilities were calculated using ProteinProphet [106] with SEQUEST results.

## Abbreviations

COG: cluster of orthologous groups; DR: direct repeat; HGT: Horizontal gene transfer; HMM: hidden Markov model; ICE: integrative and conjugative element; LC-MS/MS: capillary liquid chromatography-tandem mass spectrometry; LP: lipoprotein; *Mcc *Kid: *Mycoplasma capricolum *subsp. *capricolum *strain Kid; *Mmm *SC PG1: *Mycoplasma mycoides *subsp. *mycoides *small colony biotype strain PG1; SP: signal peptide; TM: trans-membrane; VNTR: variable nucleotide tandem repeat.

## Authors' contributions

KR designed aspects of the study, performed motif searches, genomic comparisons, assorted sequence analyses and phylogenetic studies, and helped draft the manuscript. MF performed motif searches, assorted sequence analyses, and the evaluations of sequence variation and palindromy. SY constructed HMMs, designed their applications, performed motif searches of large datasets, and constructed phylogenetic trees of prokaryotes with complete genome sequences. JG provided formative discussions leading to critical design concepts of the study and initial insights into protein structural aspects. MC provided key initial insights into sequence repeat patterns and their distribution among genomes and mobility elements. KW conceived, coordinated and participated in the design of most studies, performed assorted sequence analyses and drafted the manuscript. All authors contributed significant intellectual content during the drafting and revision of the manuscript and read and approved the submitted version.

## Supplementary Material

Additional file 1 **HMM domains in the non-redundant protein sequence database**. HMM-defined domains in the nr database are listed by taxonomic distribution and parsed by ORF (sheet 1), or as individual domains in the ORFs with their corresponding sequences (sheet 2). Click here for file

Additional file 2 **HMM domains in the non-redundant environmental protein sequence database**. HMM-defined domains in the nr environmental database are listed by ORF (sheet 1) or as individual domains in the ORFs with their corresponding sequences (sheet 2). Click here for file

Additional file 3 **Phylogenetic distribution of the HMM motif among all bacteria**. A neighbor-joining distance tree based on 16 S rRNA gene sequences depicts the bacterial taxa found to harbor the HMM motif. Major phyla and some pertinent subgroups are indicated on the right. The tree was constructed as described in Methods. Bootstrap support values above 50% are shown. Click here for file

Additional file 4 **Sporadic distribution of the HMM motif within two disparate groups: unicellular eucaryotes and wall-less bacteria**. Neighbor-joining distance trees based on small-subunit rRNA gene sequences were constructed as described in Methods to illustrate the punctate pattern of distribution characteristic of the HMM motif, using different examples from the eucaryotes, and the monoderm bacteria *Mollicutes*. (A) A tree based on 18 S rRNA gene sequences depicts selected eucaryotic taxa from diverse phylogenetic clades, including all taxa found to harbor the HMM motif (red font). The number of motif-containing ORFs annotated in the respective genome is indicated in brackets. Major eucaryotic groups [28,29] are indicated on the right. Bootstrap support values above 50% are shown. (B) A tree based on 16 S rRNA gene sequences depicts all *Mollicutes *whose genomes have been fully sequenced and assembled. Taxa harboring the HMM motif are indicated in red font. The number of motif-containing ORFs annotated in the respective genome is indicated in brackets. Major mycoplasmal sub-groups are indicated on the right. Bootstrap support values above 50% are shown. Click here for file

Additional file 5 **Distribution of HMM domains among fully sequenced and assembled genomes of Bacteria and Archaea**. A 16 S rRNA neighbor-joining phylogenetic tree depicts 891 bacterial and archaeal taxa having completely sequenced and assembled genomes. Archea and major groups of bacteria are indicated by shading. Taxa with genomes that contain annotated ORFs encoding HMM-defined domains are denoted by red font and peripheral markers. Click here for file

Additional file 6 **Representative palindromic motifs associated with PARCEL coding regions and CRISPR repeats**. (Sheet 1) DNA sequences of PARCEL-containing ORFs from several representative sources were analyzed for the occurrence of palindromic motifs as described in Methods. The derived sequences and features of palindrome motifs that correspond to the canonical HMM-defined repeat pattern are described, along with their locations relative to the protein sequence repeats. (Sheet 2) Similar analysis of the DNA sequence from a CRISP repeat region is described, showing the correspondence of derived palindromic motifs with the repeats known to have dyad symmetry. Click here for file

Additional file 7 **Features of PARCEL-containing ORFs in the genome of *Mcc *Kid**. PARCEL-containing ORFs encoded in this genome are listed by locus tags and categorized by properties denoting membrane proteins, association with mobile elements and other features. Click here for file

Additional file 8 **LC-MS/MS analysis of peptides confirming expression of PARCEL-containing LPs of *Mcc *Kid**. LC-MS/MS data describe the characteristics of tryptic peptides derived from four expressed LPs containing PARCEL domains, including peptides representing those domains. Click here for file

Additional file 9 **Description of the profile HMM defining the PARCEL protein motif**. Parameters describing the HMM that are useful for its reconstruction are displayed. Click here for file
