# Supplementary figures and images for: A versatile palindromic amphipathic repeat coding sequence horizontally distributed among diverse bacterial and eucaryotic microbes
Source: BMC Genomics. 2010 Jul 13;11:430. doi: 10.1186/1471-2164-11-430 (PMC2996958; doi:10.1186/1471-2164-11-430)

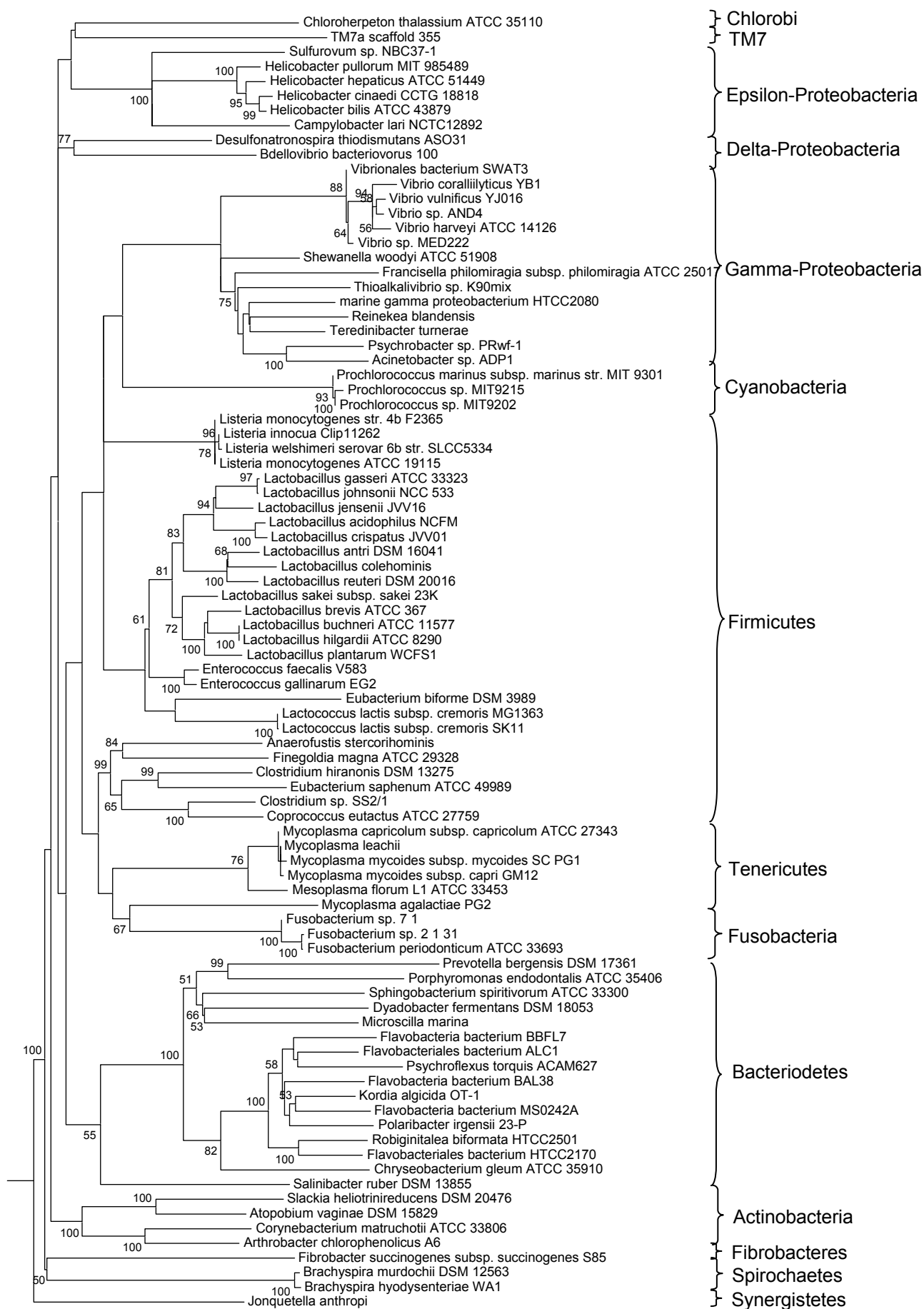

Supplement: Additional file 3 — Phylogenetic distribution of the HMM motif among all bacteria. A neighbor-joining distance tree based on 16 S rRNA gene sequences depicts the bacterial taxa found to harbor the HMM motif. Major phyla and some pertinent subgroups are indicated on the right. The tree was constructed as described in Methods. Bootstrap support values above 50% are shown. [file 1471-2164-11-430-S3.PDF]

**A**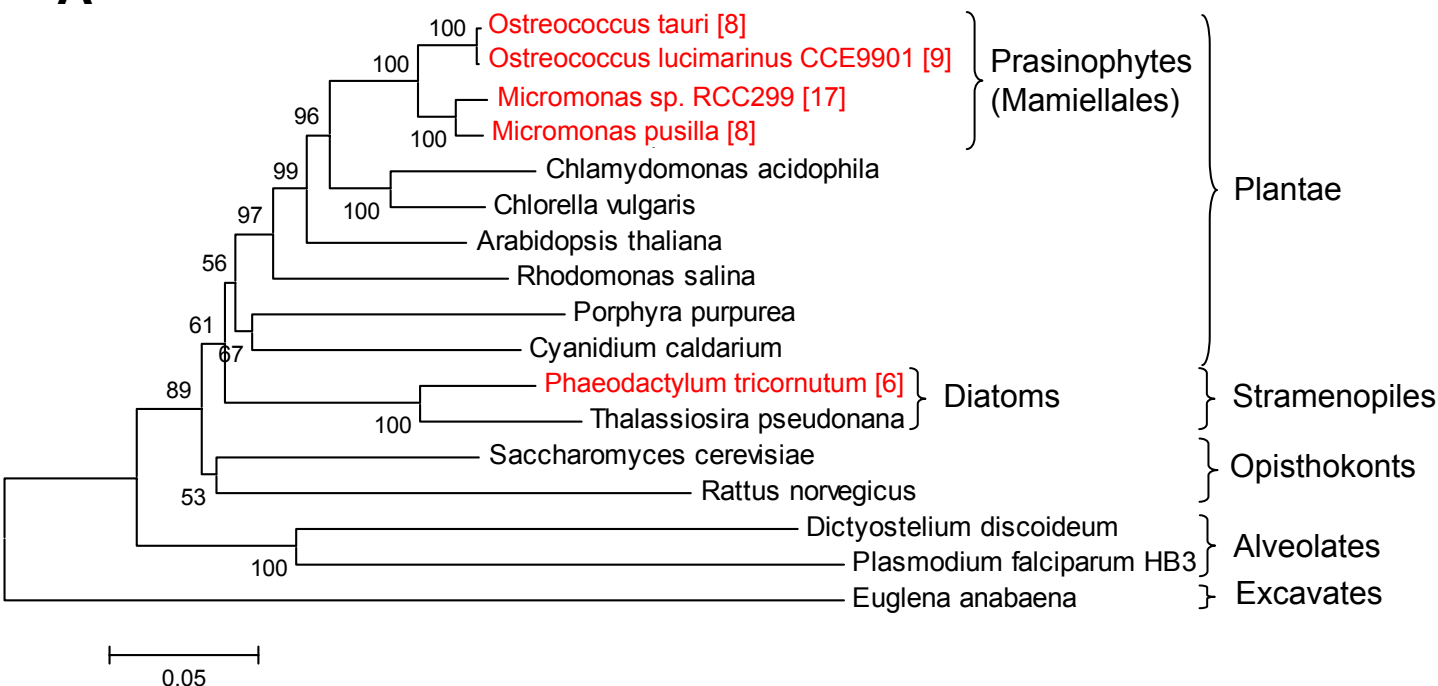**B**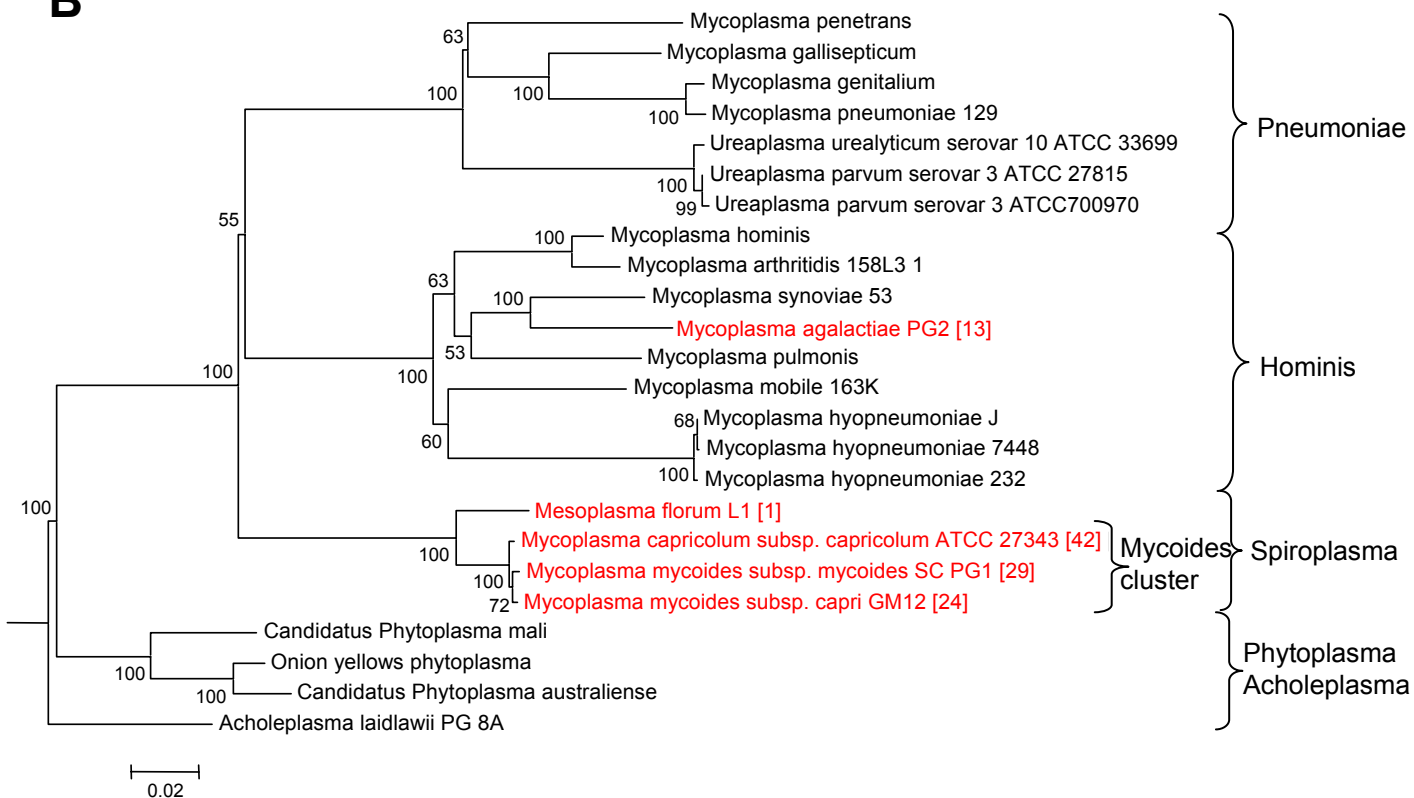

Supplement: Additional file 4 — Sporadic distribution of the HMM motif within two disparate groups: unicellular eucaryotes and wall-less bacteria. Neighbor-joining distance trees based on small-subunit rRNA gene sequences were constructed as described in Methods to illustrate the punctate pattern of distribution characteristic of the HMM motif, using different examples from the eucaryotes, and the monoderm bacteria Mollicutes. (A) A tree based on 18 S rRNA gene sequences depicts selected eucaryotic taxa from diverse phylogenetic clades, including all taxa found to harbor the HMM motif (red font). The number of motif-containing ORFs annotated in the respective genome is indicated in brackets. Major eucaryotic groups [28,29] are indicated on the right. Bootstrap support values above 50% are shown. (B) A tree based on 16 S rRNA gene sequences depicts all Mollicutes whose genomes have been fully sequenced and assembled. Taxa harboring the HMM motif are indicated in red font. The number of motif-containing ORFs annotated in the respective genome is indicated in brackets. Major mycoplasmal sub-groups are indicated on the right. Bootstrap support values above 50% are shown. [file 1471-2164-11-430-S4.PDF]
