# Supplementary material for: A versatile palindromic amphipathic repeat coding sequence horizontally distributed among diverse bacterial and eucaryotic microbes
Source: BMC Genomics. 2010 Jul 13;11:430. doi: 10.1186/1471-2164-11-430 (PMC2996958; doi:10.1186/1471-2164-11-430)
Supplement: Additional file 5 — Distribution of HMM domains among fully sequenced and assembled genomes of Bacteria and Archaea. A 16 S rRNA neighbor-joining phylogenetic tree depicts 891 bacterial and archaeal taxa having completely sequenced and assembled genomes. Archea and major groups of bacteria are indicated by shading. Taxa with genomes that contain annotated ORFs encoding HMM-defined domains are denoted by red font and peripheral markers. [file 1471-2164-11-430-S5.PDF]

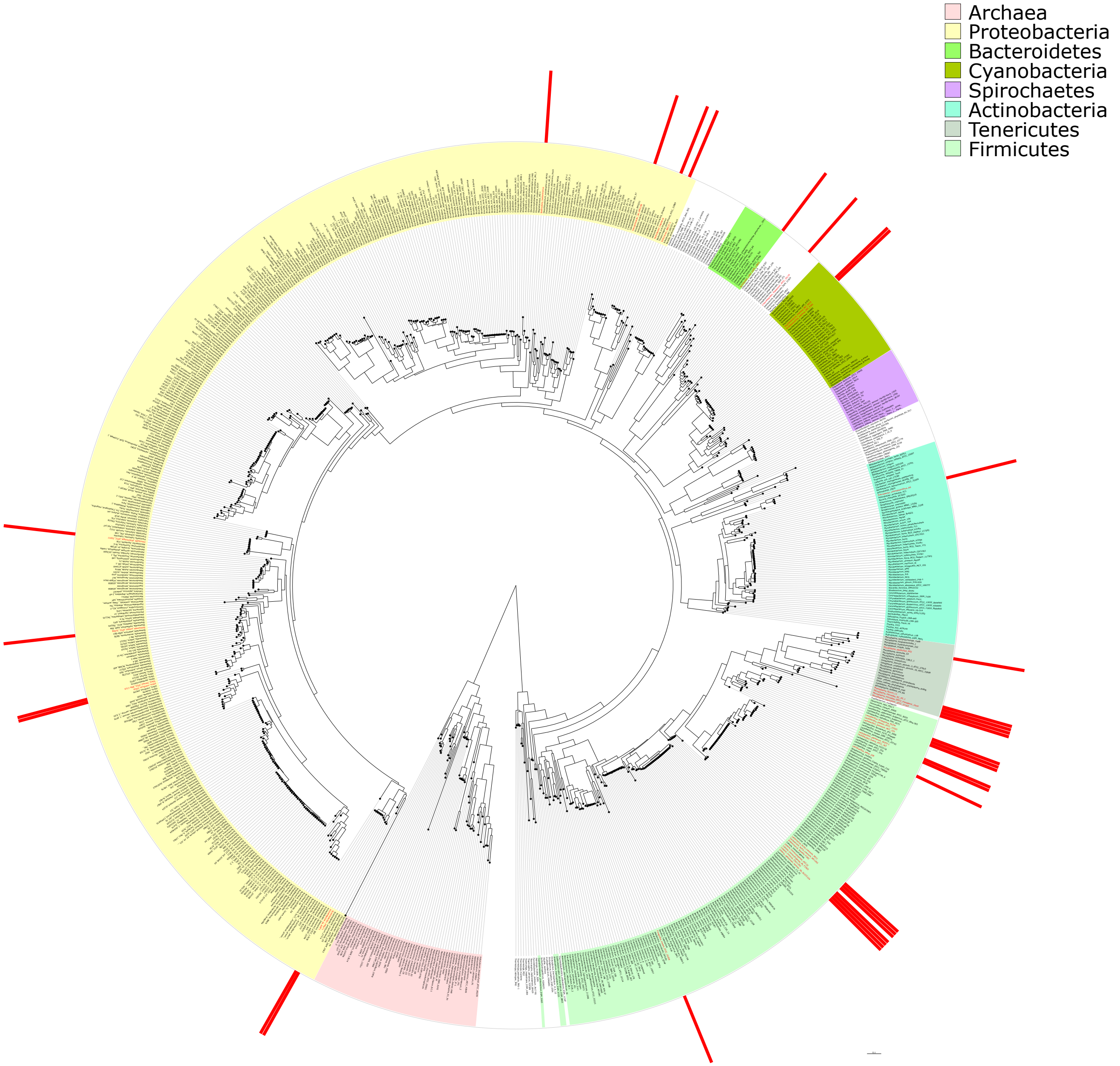
Archaea
Proteobacteria
Bacteroidetes
Cyanobacteria
Spirochaetes
Actinobacteria
Tenericutes
Firmicutes
